# Supplementary material for: Expression of the Reverse Transcriptase Domain of Telomerase Reverse Transcriptase Induces Lytic Cellular Response in DNA-Immunized Mice and Limits Tumorigenic and Metastatic Potential of Murine Adenocarcinoma 4T1 Cells
Source: Vaccines (Basel). 2020 Jun 18;8(2):318. doi: 10.3390/vaccines8020318 (PMC7350266; doi:10.3390/vaccines8020318)
Supplement: Supplementary file 1 [file vaccines-08-00318-s001.pdf]

vaccines

Supplementary materials

Supplementary Figure S1. Lentiviral vector pRRLSIN.cPPT.PGK used for transduction of 4T1luc2 cell line. Position of insertion of rtTERT gene marked in red. PGK-seq and LVT-200R (Supplementary Table 1) PCR primers were used to confirm the insertion of lentiviruses encoding rtTERT into genome of 4T1luc2 cells.

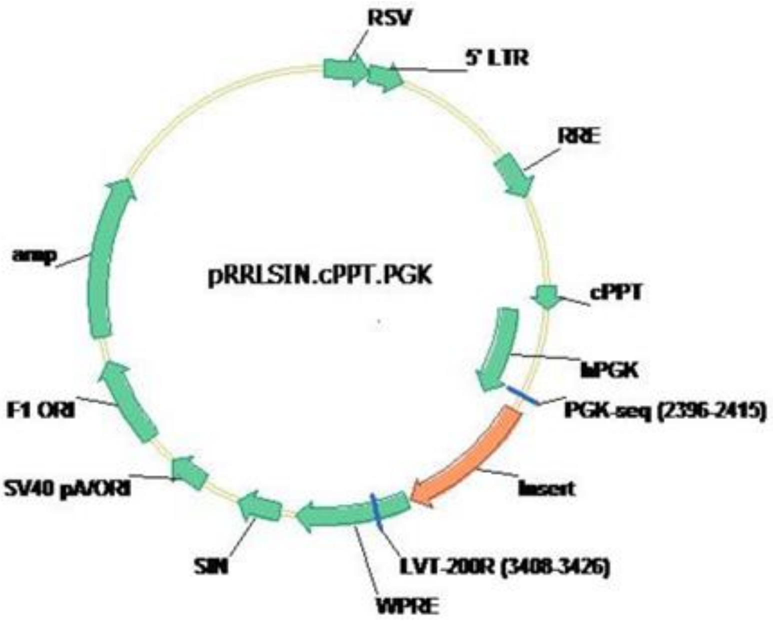

Supplementary Figure S2A-C. Results of primers validation. Two clearly distinguishable clusters of positive and negative droplets for Actb (**A**), Mstn (**B**), Tert (**C**); copy number of Mstn gene in relation to Actin B in 4T1luc2 and daughter clones (**D**).

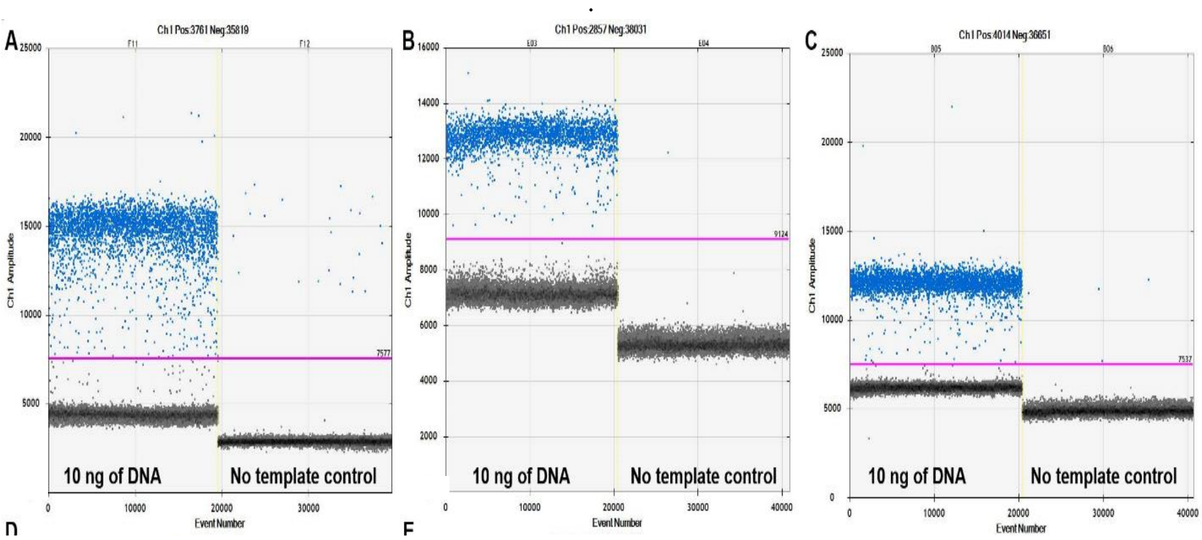

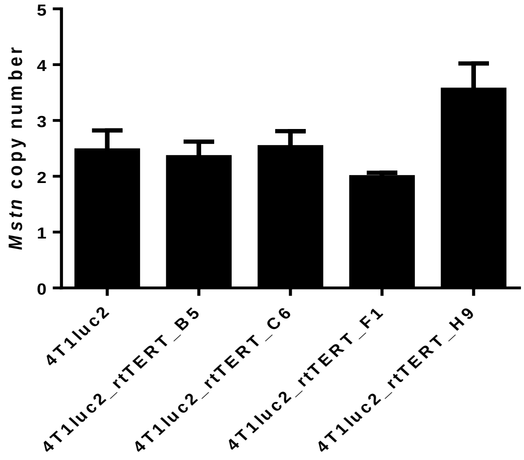

D

Supplementary Figure S3. Gating of live and single cells. Green – G1/G0 phase, yellow – S phase, turquoise – G2/M phase.

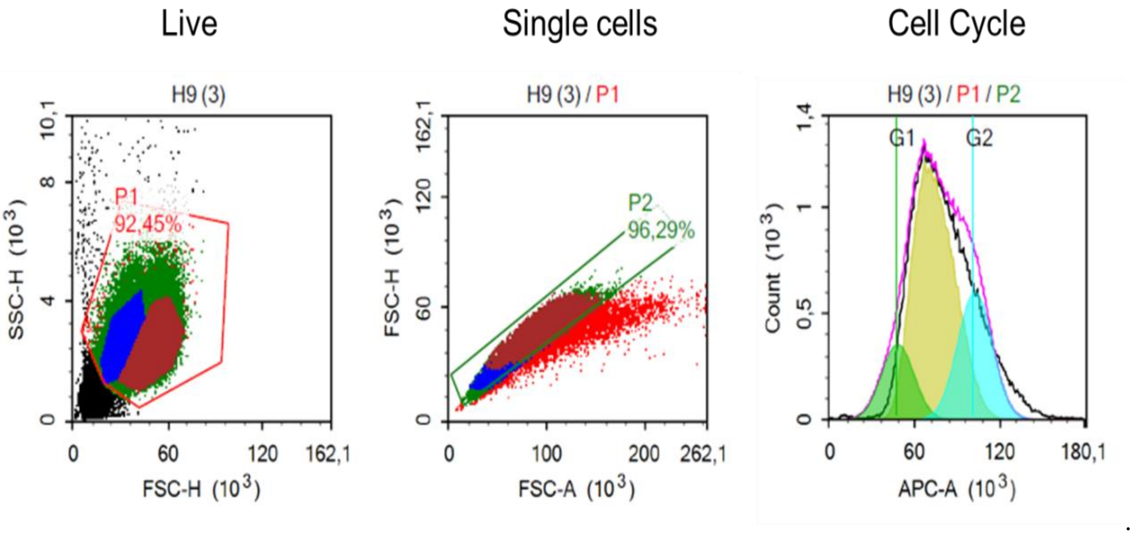

.

Supplementary Figure S4. Histograms of cells in different cell cycle phases. Green – G1/G0, yellow – S, turquoise – G2/M. Three replicates for each cell line.

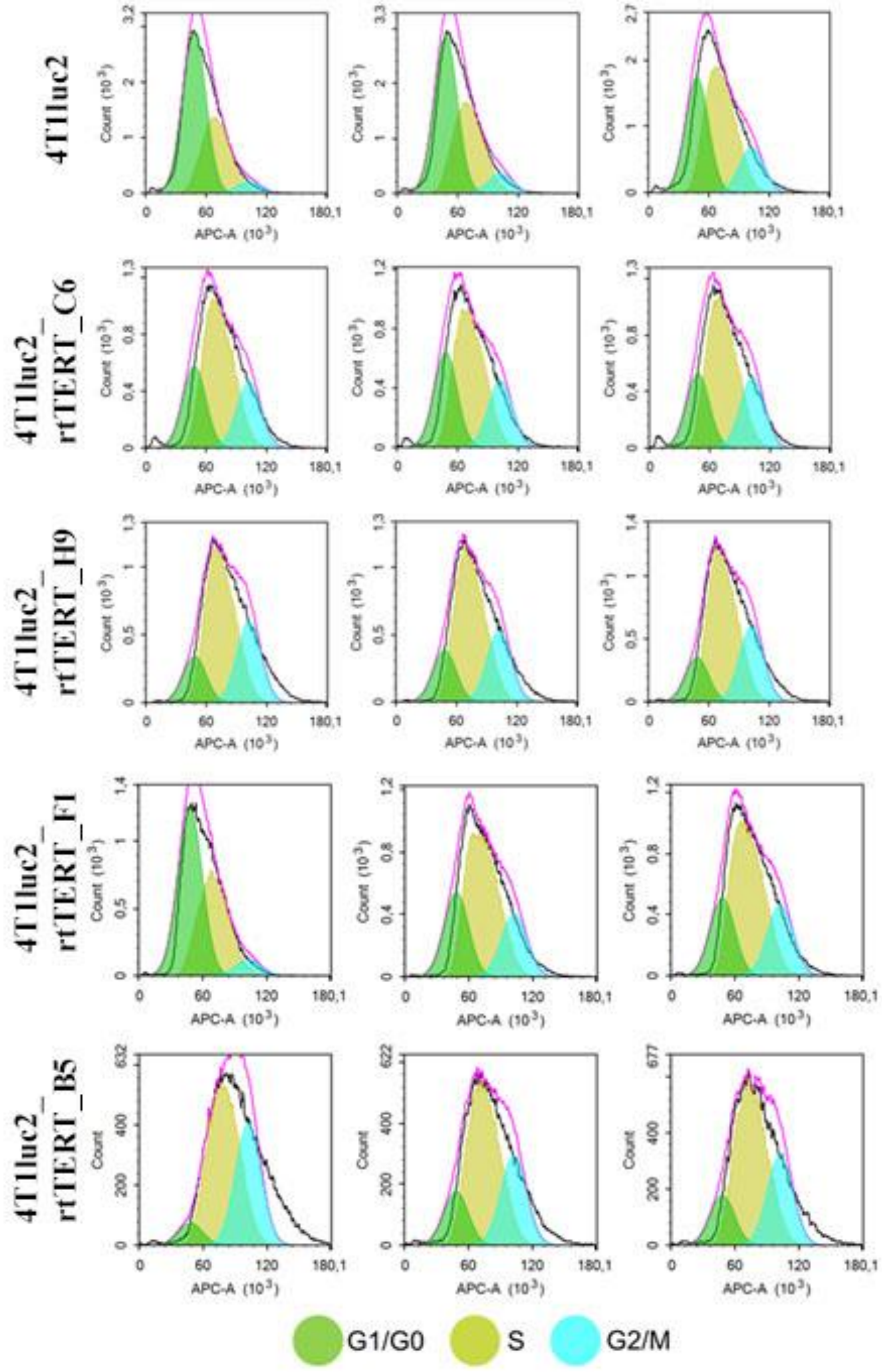

Supplementary Figure S5. The example of the multiparametric flow cytometry analysis of the splenocytes stimulated by the incubation in the presence of peptide TERT8 inducing activation of both helpers and cytotoxic T-lymphocytes. The size of IFN-γ, double IFN-γ/IL-2 and triple IFN-γ/IL-2 /TNF-α-expressing cells populations are shown as proportion of respectivr cytokine-producing CD4+ or CD8+ cells.

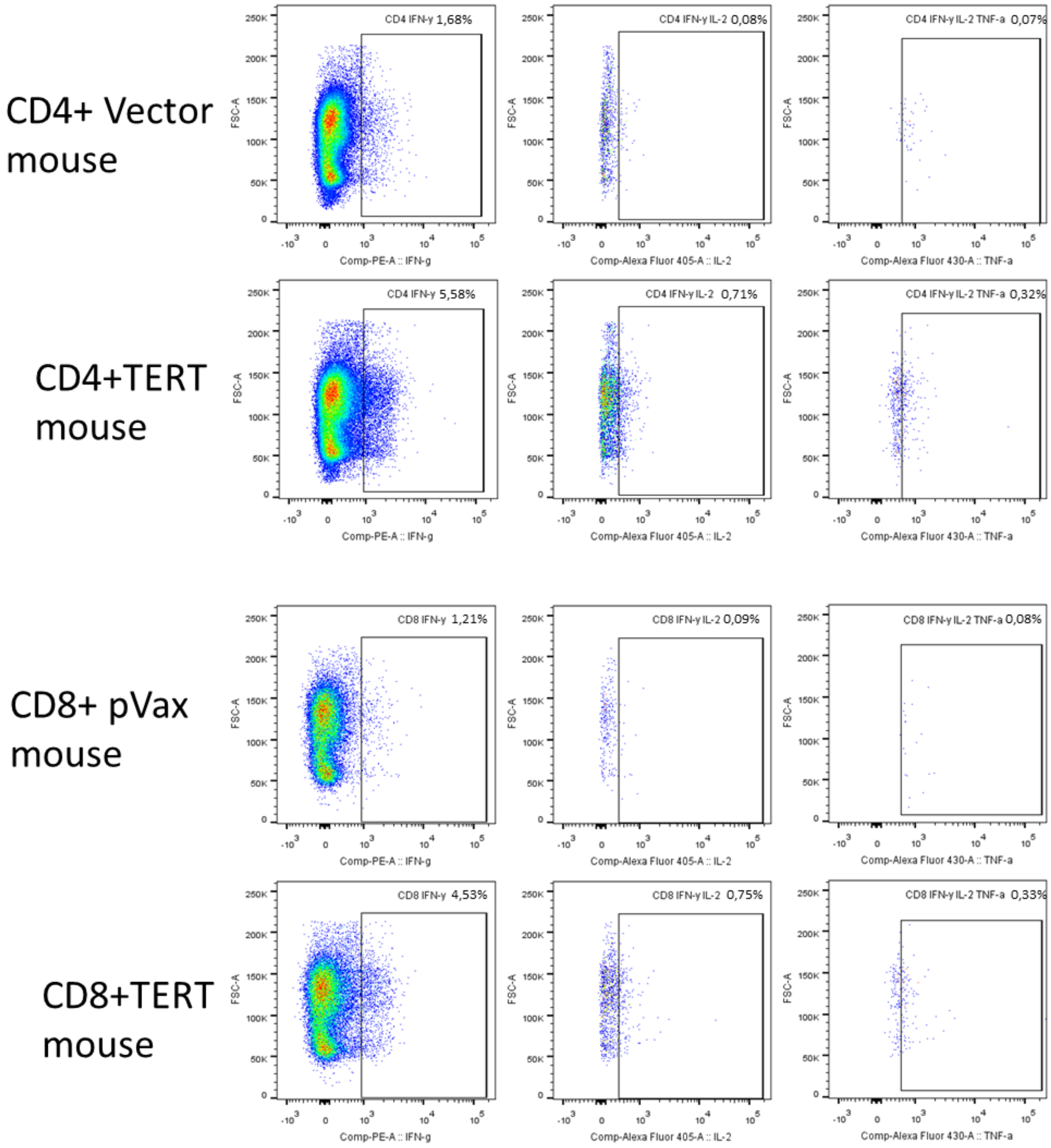

Supplementary Figure S6. Expression of rat TERT in 293Tcells transiently transfected with rat TERT-encoding plasmids. Western blotting of the lysates of 293Tcells transfected with vector pVAX1 (lane 1), pVax-TERT (lane 2), and pVax-TERT-HA (lane 3). Blots were stained with commercial rabbit polyclonal anti-TERT antibodies raised against synthetic peptide (**A**), and re-stained with monoclonal anti-actin antibodies (**B**). Positions of molecular mass markers are given to the right in kDa.

A

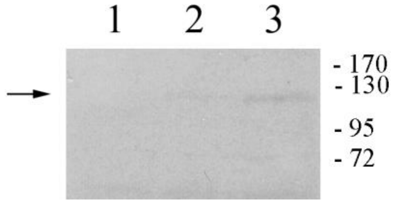

B

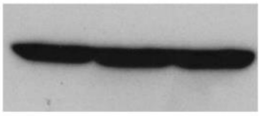

Supplementary Figure S7. Immune recognition of TERT-derived peptides (Table 1) by CD4+ and CD8+ T cells of mice DNA-immunized with TERT or TERT-HA compared to vector-immunized mice analyzed by multiparametric flow cytometry. Percent of CD4+ (**A**) and CD8+ (**B**) T cells reacting to stimulation with TERT peptides bysingle cytokine expression. Values represent mean±SD. Difference between TERT, TERT-HA DNA-immunized and control vector-immunized mice was analyzed by Mann-Whitney test. Difference between TERT, TERT-HA DNA-immunized and control vector *- $p < 0.05$; **- $p < 0.01$. No difference betwee TERT and TERT-HA DNA-immunized mice weas found, all $p > 0.05$.

A

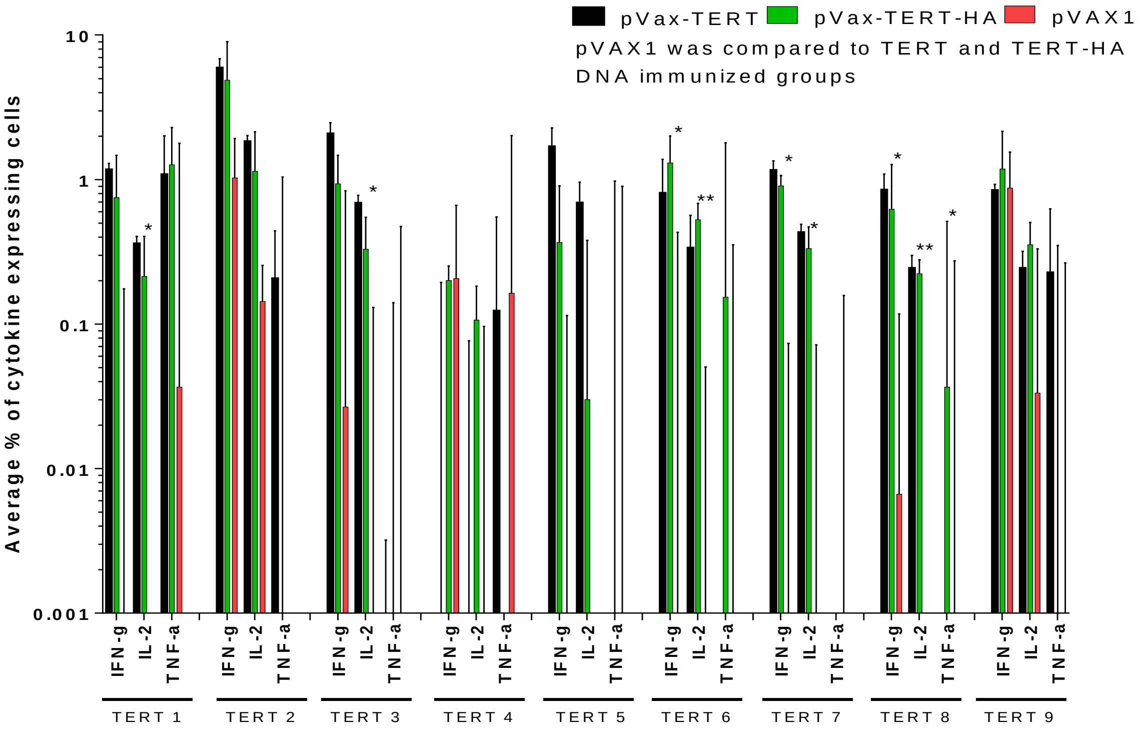

B

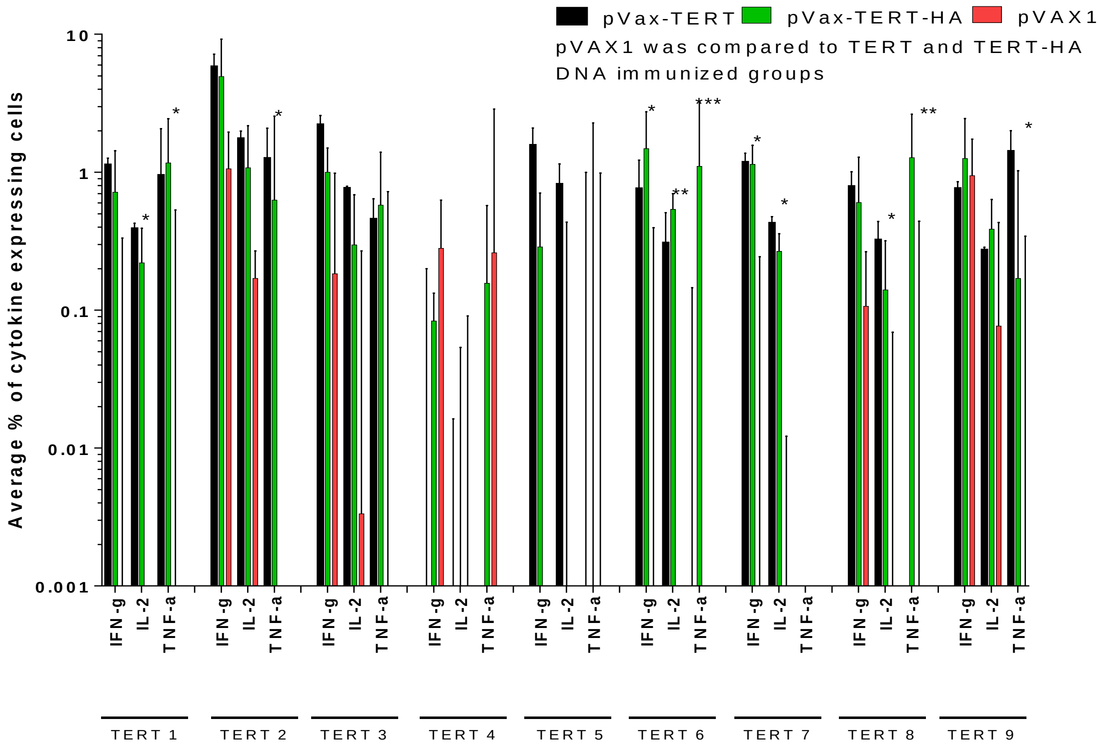

Supplementary Figure S8. Correlates of the number of metastases in liver of mice implanted with 4T1luc2 cells expressing rtTERT. Average number of liver metastases correlates with max radiance from liver in *ex vivo* organ imaging (R = 0.5; *p* = 0.024) (**A**); average number of liver metastases and their size correlates to the number of liver-infiltrating leukocytes (R = 0.83, **p** < 10^{-4}) (**B**).

A

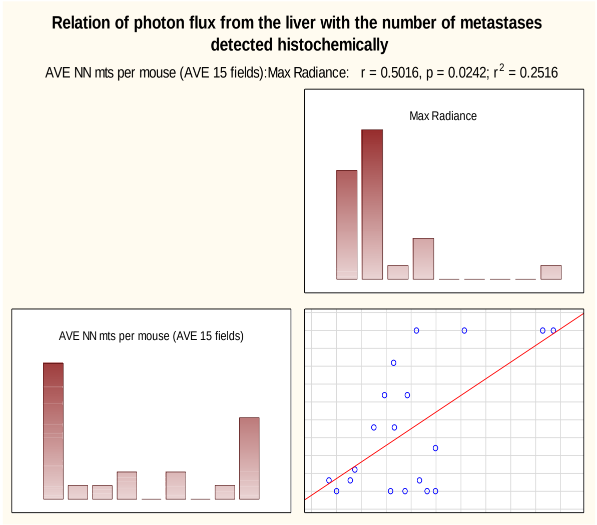

B

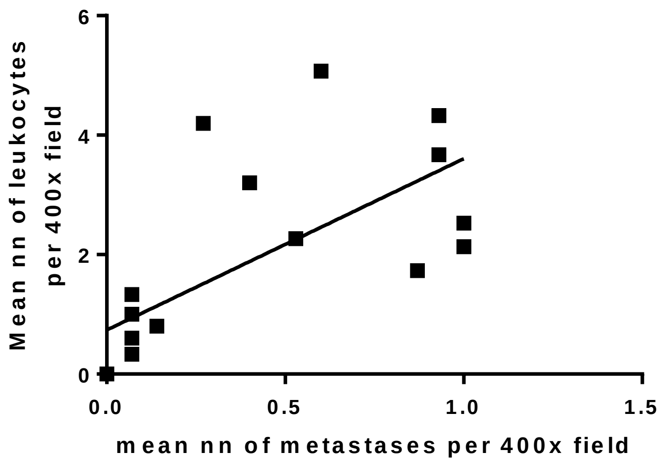

Supplementary Figure S9. Comparison of the overall profile of *in vitro* cytokine response of CD4+ (**A, C, E**) and CD8+ (**B, D, F**) T cells of mice implanted with rtTERT expressing cell lines 4T1luc2_rtTERT_H9 (H9; n = 4) and 4T1luc2_rtTERT_C6 (C6; n = 4) and parental cell line (4T1luc2; n = 4) to stimulation with TERT peptides: TERT2 presenting an autoepitope (A, B); TERT6 (C, D); and TERT8 (E, F). Cytokine production was assessed by multiparametric flow cytometry and presented as percent of mono-, di- and tri-cytokine producing CD4+ and CD8+ T cells. Statistical analysis was performed by Friedman ANOVA test and Kendall Coefficeint of concordance (Statistica AXA 11.0). Criteria of statistically significant difference were set at $p < 0.01$.

A

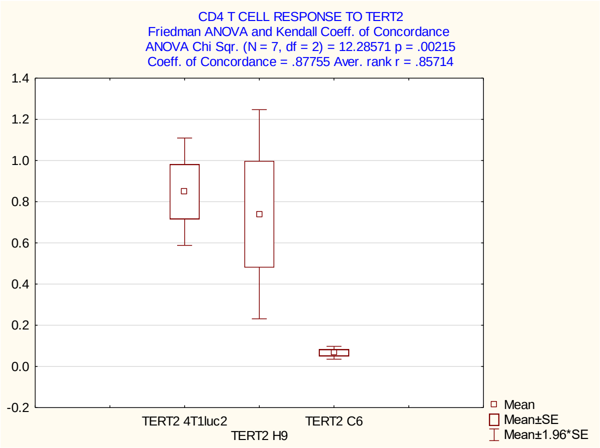

B

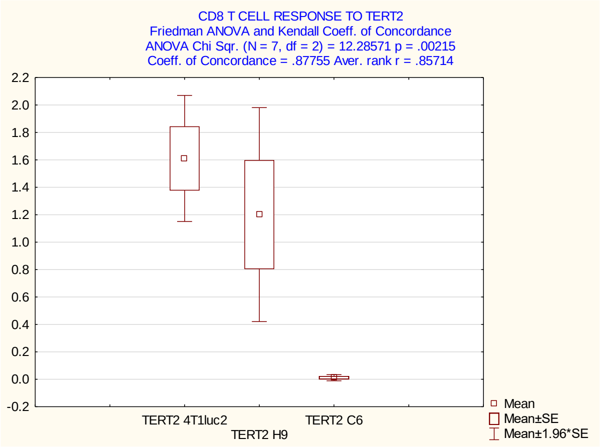

C

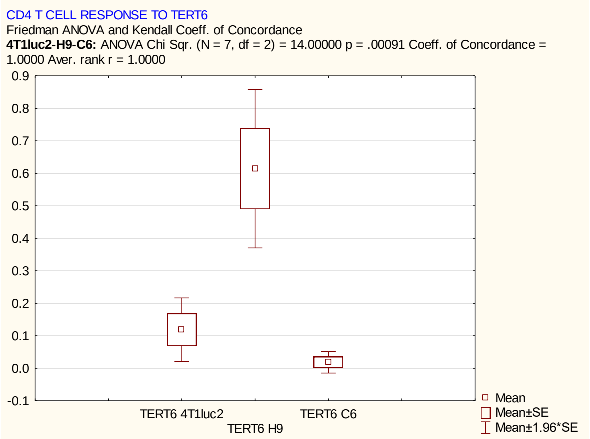

D

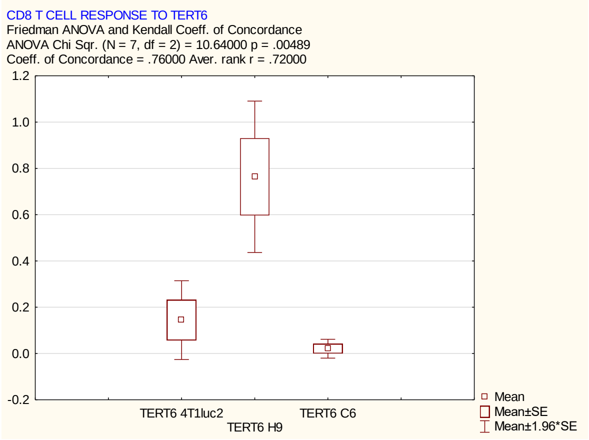
CD8 T CELL RESPONSE TO TERT6
Friedman ANOVA and Kendall Coeff. of Concordance
ANOVA Chi Sqr. (N = 7, df = 2) = 10.64000 p = .00489
Coeff. of Concordance = .76000 Aver. rank r = .72000
1.2
1.0
0.8
0.6
0.4
0.2
0.0
-0.2
TERT6 4T1luc2
TERT6 H9
TERT6 C6
Mean
Mean±SE
Mean±1.96*SE

E

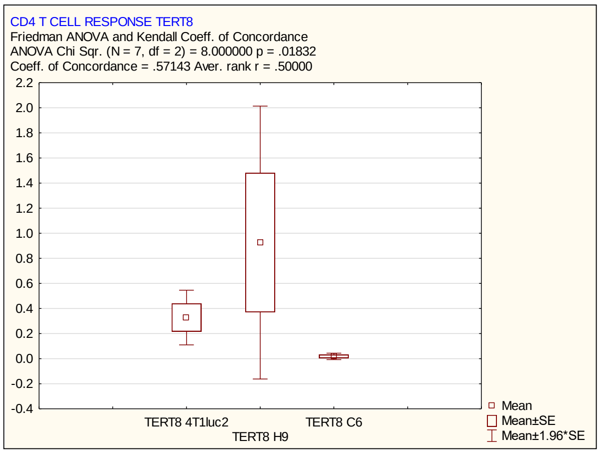
CD4 T CELL RESPONSE TERT8
Friedman ANOVA and Kendall Coeff. of Concordance
ANOVA Chi Sqr. (N = 7, df = 2) = 8.000000 p = .01832
Coeff. of Concordance = .57143 Aver. rank r = .50000
2.2
2.0
1.8
1.6
1.4
1.2
1.0
0.8
0.6
0.4
0.2
0.0
-0.2
-0.4
TERT8 4T1luc2
TERT8 H9
TERT8 C6
Mean
Mean±SE
Mean±1.96*SE

F

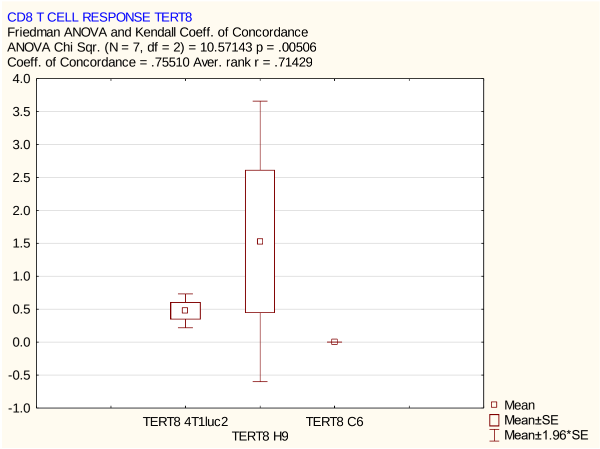
CD8 T CELL RESPONSE TERT8
Friedman ANOVA and Kendall Coeff. of Concordance
ANOVA Chi Sqr. (N = 7, df = 2) = 10.57143 p = .00506
Coeff. of Concordance = .75510 Aver. rank r = .71429
4.0
3.5
3.0
2.5
2.0
1.5
1.0
0.5
0.0
-0.5
-1.0
TERT8 4T1luc2
TERT8 H9
TERT8 C6
Mean
Mean±SE
Mean±1.96*SE

Supplementary Figure S10. Overall profile of in vitro cytokine response of CD4+ (A) and CD8+ (B) T cells of mice implanted with rtTERT expressing cell lines 4T1luc2_rtTERT_H9 (H9; n = 4) and 4T1luc2_rtTERT_C6 (C6; n = 4) and parental cell line (4T1luc2; n = 4) to stimulation with mitogen PMA. Cytokine production was assessed by multiparametric flow cytometry and presented as percent of mono-, di- and tri-cytokine-producing CD4+ and CD8+ T cells. Statistical analysis of the profile for CD4+ (C) and CD8+ T cells (D) was performed by Friedman ANOVA test and Kendall Coefficeint of concordance (Statistica AXA 10.0); $p < 0.01$ was considered as significant.

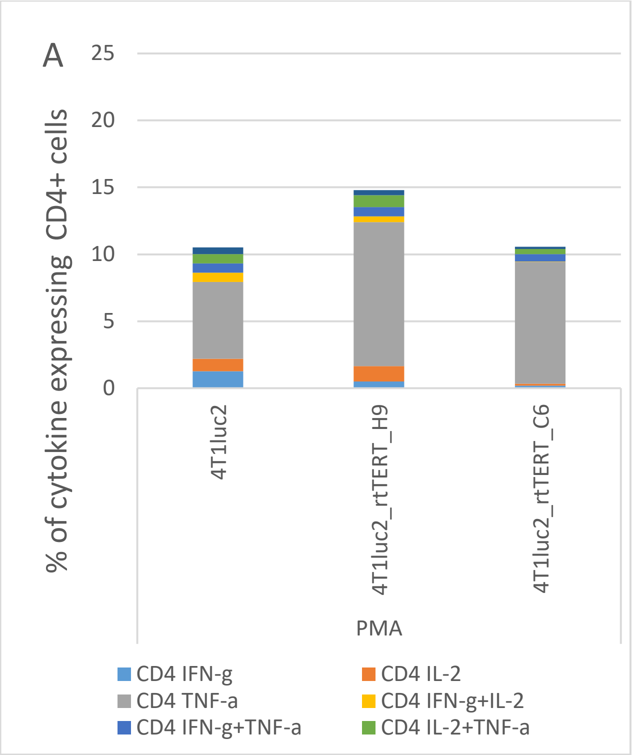

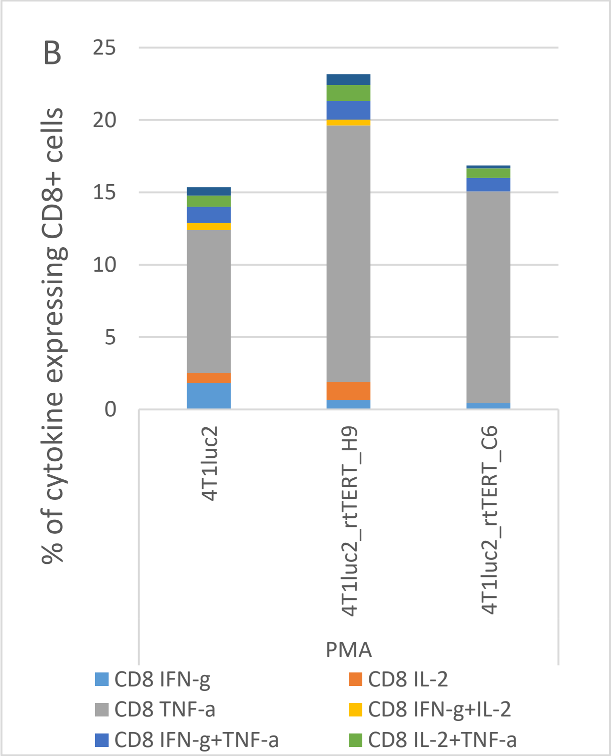

C

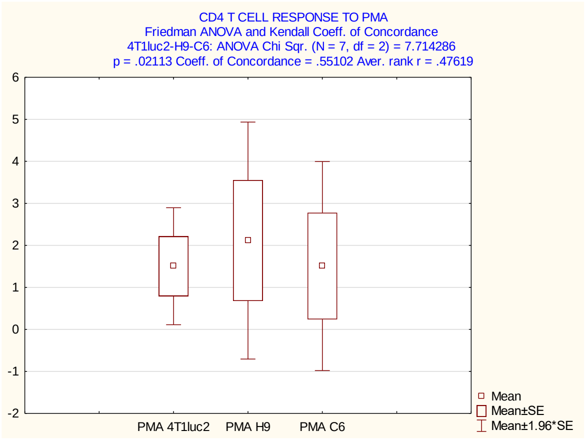
CD4 T CELL RESPONSE TO PMA
Friedman ANOVA and Kendall Coeff. of Concordance
4T1luc2-H9-C6: ANOVA Chi Sqr. (N = 7, df = 2) = 7.714286
p = .02113 Coeff. of Concordance = .55102 Aver. rank r = .47619
6
5
4
3
2
1
0
-1
-2
PMA 4T1luc2
PMA H9
PMA C6
Mean
Mean±SE
Mean±1.96*SE

D

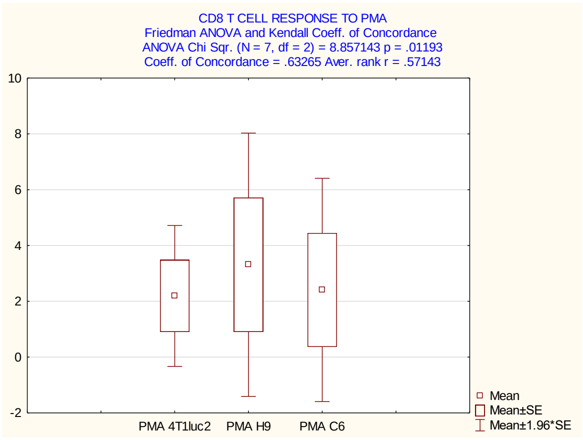
CD8 T CELL RESPONSE TO PMA
Friedman ANOVA and Kendall Coeff. of Concordance
ANOVA Chi Sqr. (N = 7, df = 2) = 8.857143 p = .01193
Coeff. of Concordance = .63265 Aver. rank r = .57143
10
8
6
4
2
0
-2
PMA 4T1luc2
PMA H9
PMA C6
Mean
Mean±SE
Mean±1.96*SE

D)

Supplementary Figure S11. Features of anti-TERT immune response promoting and restricting tumor growth. Percent of IFN/g/IL-2 secreting CD4+ (**A, C**) and CD8+ (**B, D**) T cells specific to TERT 2 (A, B) and TERT1, TERT6 and TERT8 (TERT168, summed) (C, D) in mice with aggressively growing tumors (n = 4), non aggressive tumors (restricted tumor growth, n = 4) and no tumors (rejected tumors or tumors <10 mm^3, n = 4); Correlation of the tumor size (mm2) by experimental end-point to the percent of TERT2- specific CD4 (**E**) and CD8 (**F**) T cells secreting one, two or three cytokines; Correlation of tumor growth as photon fluz from tumor implantation site by day 16 to percent of TNF-a secreting CD4+ positive T cells specific to TERT2 (**G**, n = 8). Multiple comparisons are done using Kruskall Wallis, and pair-wise, using Mann Whitney test; correlations, using Spearman ranking test (Statistica Axa 11), all p values <0.05 are cosndiered as signficant, and $p < 0.1$ as tendency to a difference.

A

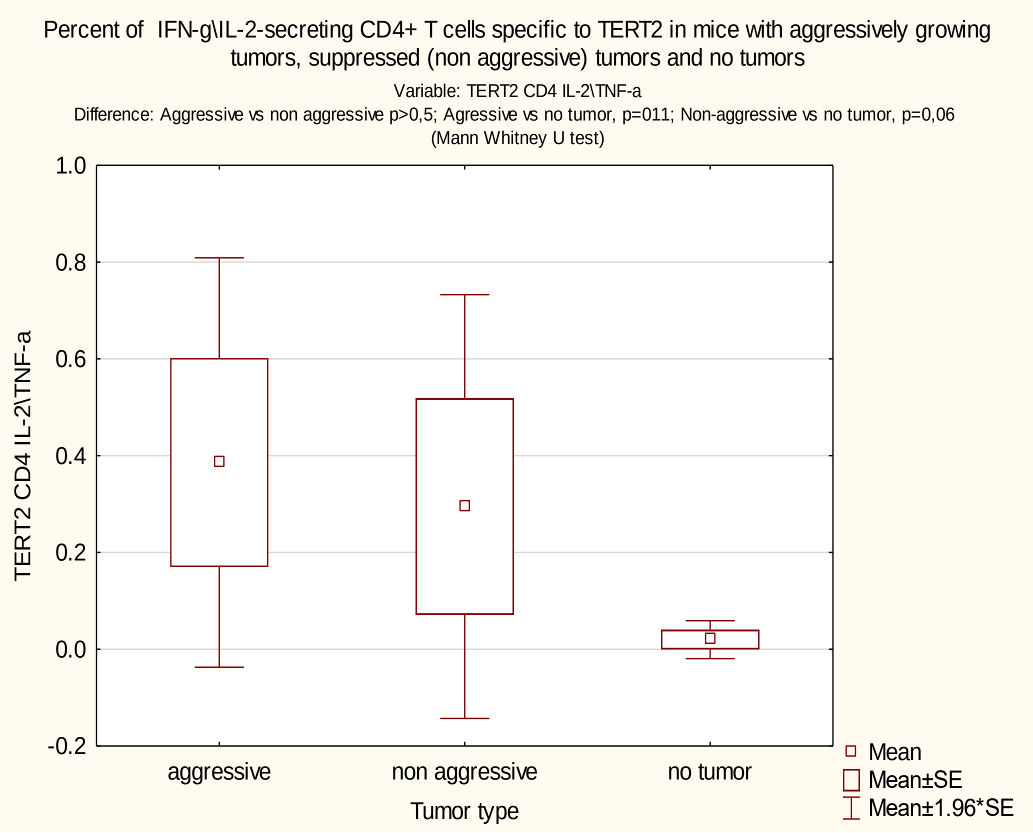

B

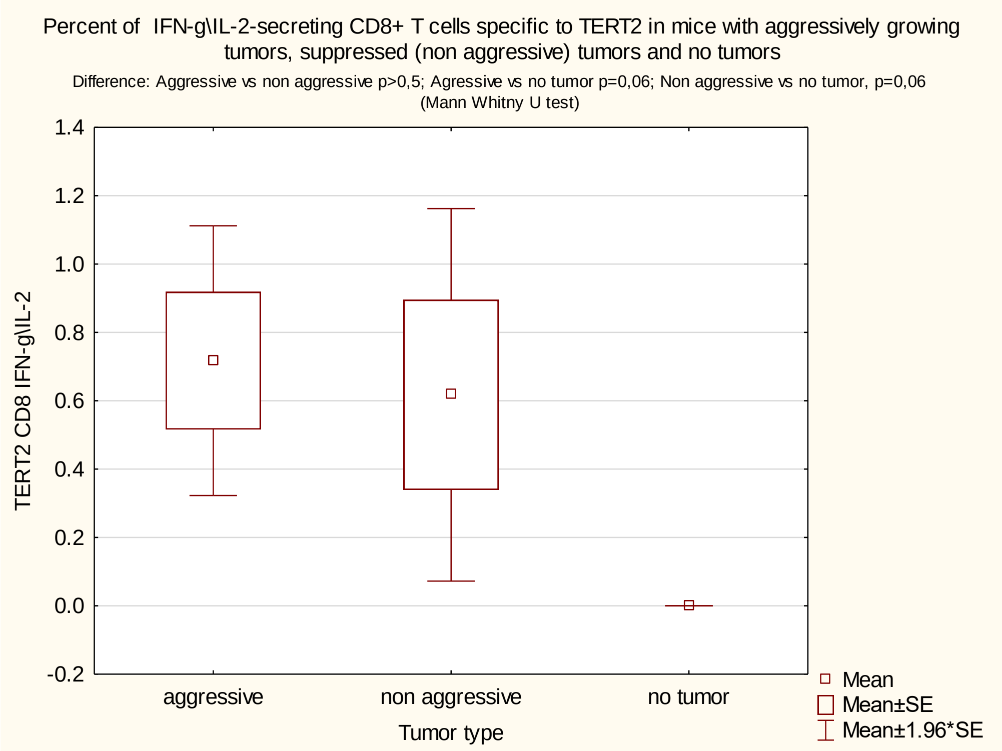
Percent of IFN-g\IL-2-secreting CD8+ T cells specific to TERT2 in mice with aggressively growing tumors, suppressed (non aggressive) tumors and no tumors
Difference: Aggressive vs non aggressive p>0,5; Agressive vs no tumor p=0,06; Non aggressive vs no tumor, p=0,06
(Mann Whitny U test)
TERT2 CD8 IFN-g\IL-2
1.4
1.2
1.0
0.8
0.6
0.4
0.2
0.0
-0.2
aggressive
non aggressive
no tumor
Tumor type
Mean
Mean±SE
Mean±1.96*SE

C

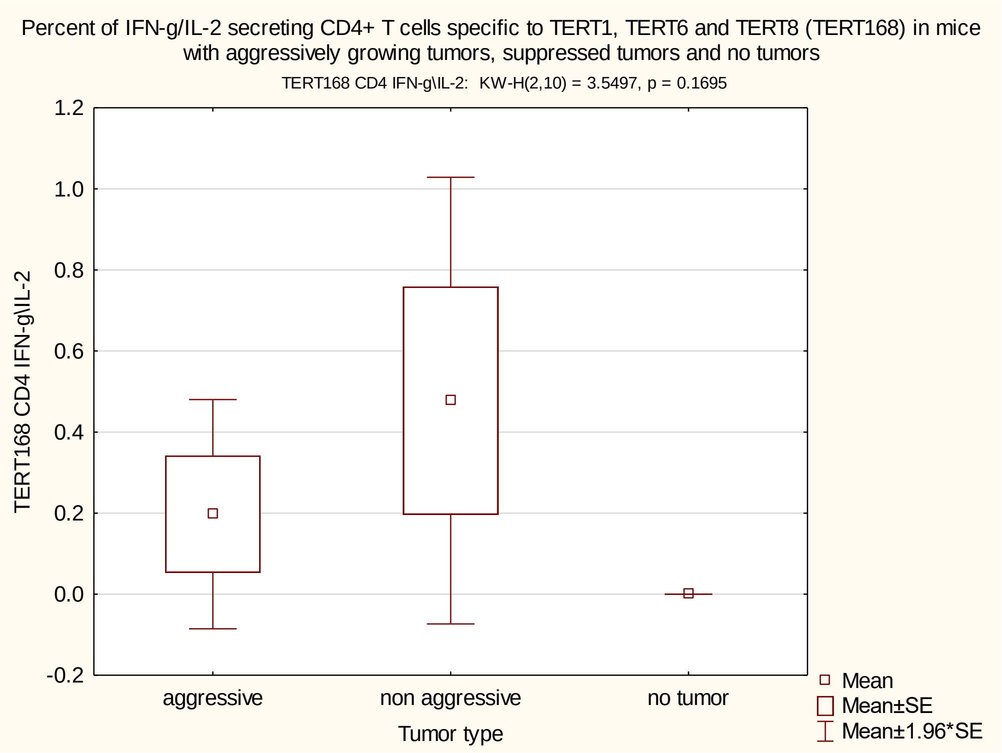
Percent of IFN-g/IL-2 secreting CD4+ T cells specific to TERT1, TERT6 and TERT8 (TERT168) in mice with aggressively growing tumors, suppressed tumors and no tumors
TERT168 CD4 IFN-g\IL-2: KW-H(2,10) = 3.5497, p = 0.1695
TERT168 CD4 IFN-g\IL-2
1.2
1.0
0.8
0.6
0.4
0.2
0.0
-0.2
aggressive
non aggressive
no tumor
Tumor type
Mean
Mean±SE
Mean±1.96*SE

D

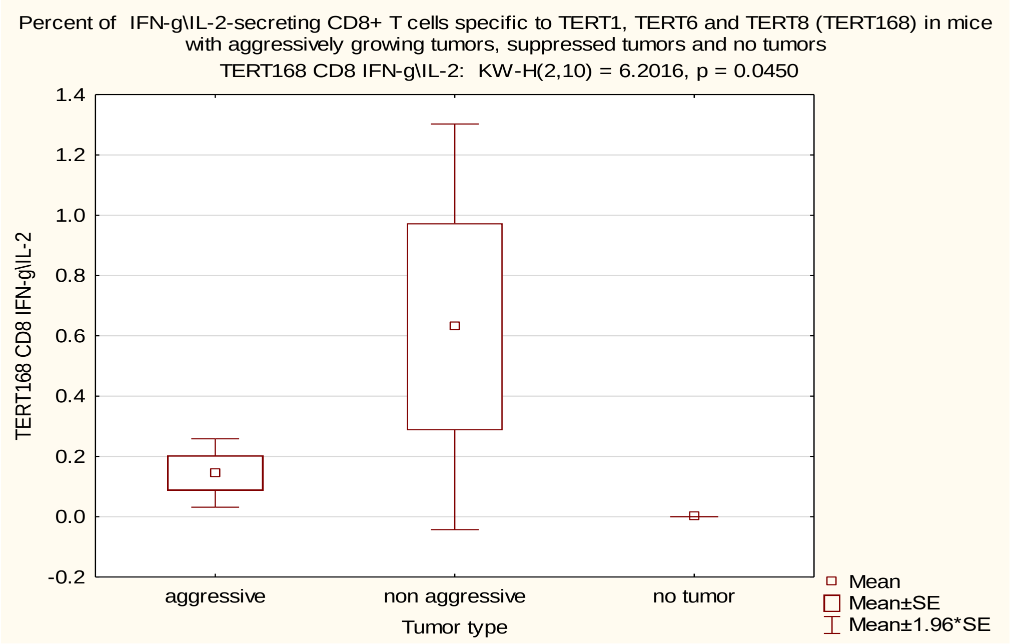

Percent of IFN-g\IL-2-secreting CD8+ T cells specific to TERT1, TERT6 and TERT8 (TERT168) in mice with aggressively growing tumors, suppressed tumors and no tumors
TERT168 CD8 IFN-g\IL-2: KW-H(2,10) = 6.2016, p = 0.0450
TERT168 CD8 IFN-g\IL-2
1.4
1.2
1.0
0.8
0.6
0.4
0.2
0.0
-0.2
aggressive
non aggressive
no tumor
Tumor type
Mean
Mean±SE
Mean±1.96*SE

E

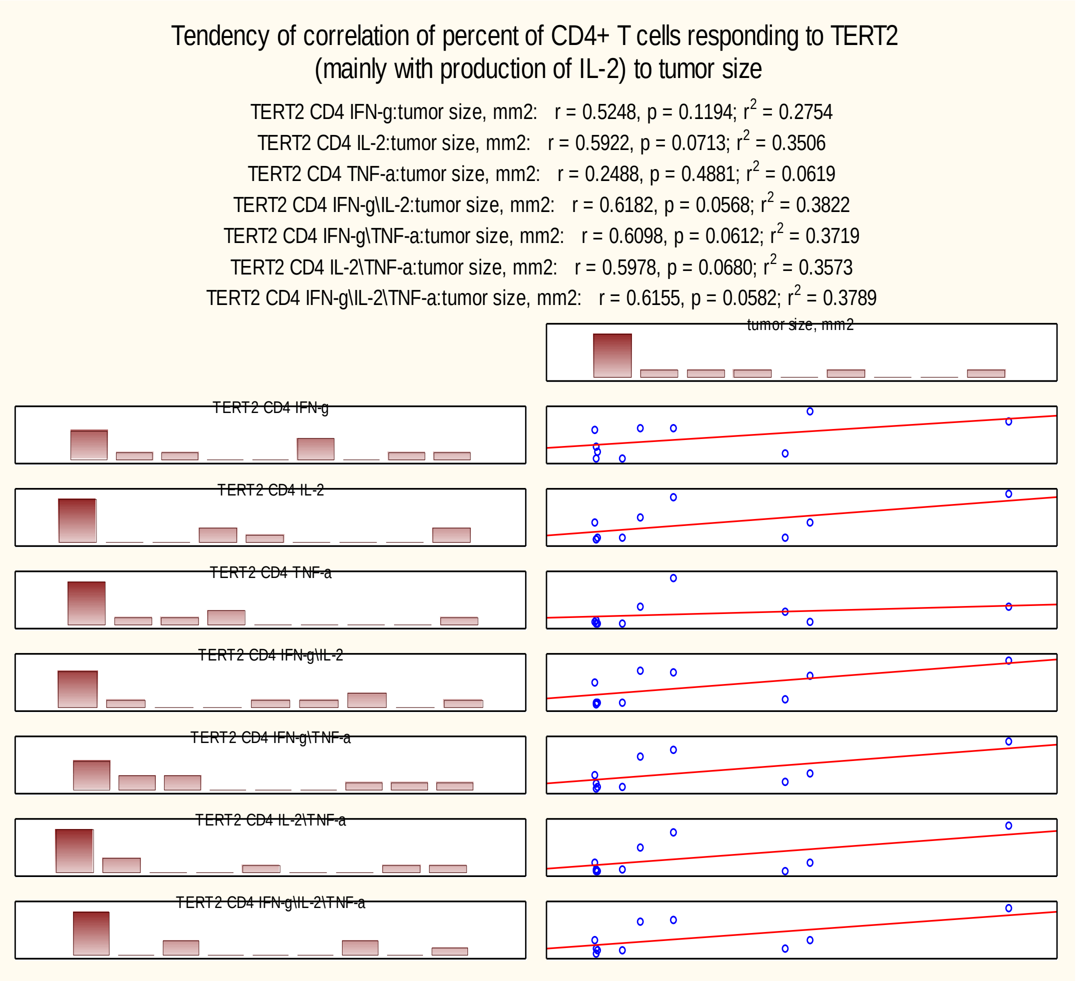

Tendency of correlation of percent of CD4+ T cells responding to TERT2 (mainly with production of IL-2) to tumor size
TERT2 CD4 IFN-g:tumor size, mm2: r = 0.5248, p = 0.1194; r2 = 0.2754
TERT2 CD4 IL-2:tumor size, mm2: r = 0.5922, p = 0.0713; r2 = 0.3506
TERT2 CD4 TNF-a:tumor size, mm2: r = 0.2488, p = 0.4881; r2 = 0.0619
TERT2 CD4 IFN-g\IL-2:tumor size, mm2: r = 0.6182, p = 0.0568; r2 = 0.3822
TERT2 CD4 IFN-g\TNF-a:tumor size, mm2: r = 0.6098, p = 0.0612; r2 = 0.3719
TERT2 CD4 IL-2\TNF-a:tumor size, mm2: r = 0.5978, p = 0.0680; r2 = 0.3573
TERT2 CD4 IFN-g\IL-2\TNF-a:tumor size, mm2: r = 0.6155, p = 0.0582; r2 = 0.3789
tumor size, mm2
TERT2 CD4 IFN-g
TERT2 CD4 IL-2
TERT2 CD4 TNF-a
TERT2 CD4 IFN-g\IL-2
TERT2 CD4 IFN-g\TNF-a
TERT2 CD4 IL-2\TNF-a
TERT2 CD4 IFN-g\IL-2\TNF-a

F

No correlation of percent of CD8+ T cells responding to TERT2
to tumor size

TERT2 CD8 IFN-g:tumor size, mm2: r = 0.0440, p = 0.8978; r^2 = 0.0019
TERT2 CD8 IL-2:tumor size, mm2: r = 0.2530, p = 0.4530; r^2 = 0.0640
TERT2 CD8 TNF-a:tumor size, mm2: r = 0.1077, p = 0.7526; r^2 = 0.0116
TERT2 CD8 IFN-g\IL-2:tumor size, mm2: r = 0.3167, p = 0.3427; r^2 = 0.1003
TERT2 CD8 IFN-g\TNF-a:tumor size, mm2: r = 0.2076, p = 0.5402; r^2 = 0.0431
TERT2 CD8 IL-2\TNF-a:tumor size, mm2: r = 0.1832, p = 0.5897; r^2 = 0.0336
TERT2 CD8 IFN-g\IL-2\TNF-a:tumor size, mm2: r = 0.2241, p = 0.5076; r^2 = 0.0502

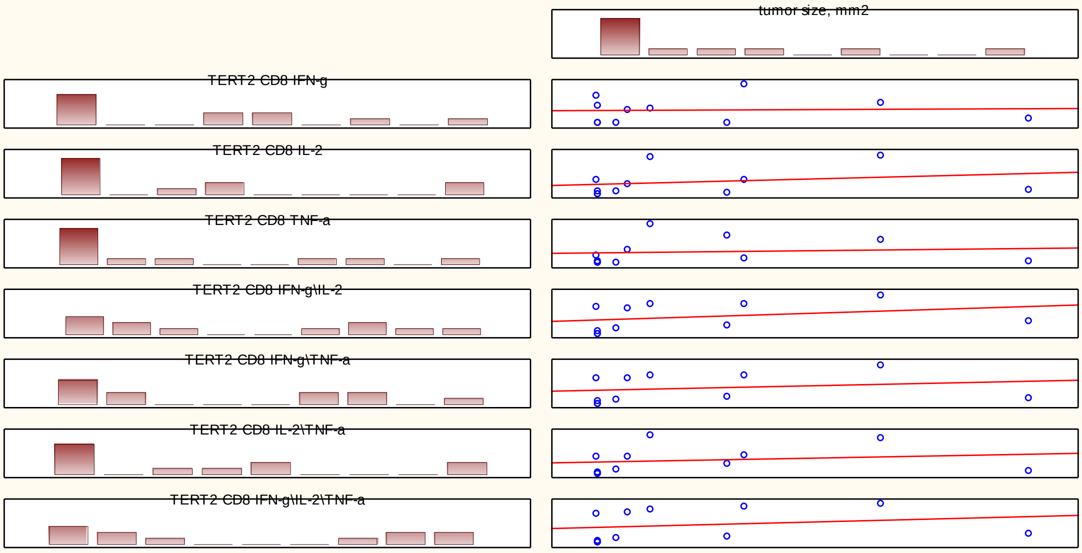

G

Correlation of tumor growth (bu photon flux) to
percent of TNF-a secreting CD4+ T cells specific to TERT2

TERT2 CD4 TNF-a:total flux, d16: r = 0.7122, p = 0.0475; r^2 = 0.5073
TERT2 CD4 IFN-g\TNF-a:total flux, d16: r = 0.6686, p = 0.0699; r^2 = 0.4470
TERT2 CD4 IFN-g\IL-2\TNF-a:total flux, d16: r = 0.6449, p = 0.0843; r^2 = 0.4158

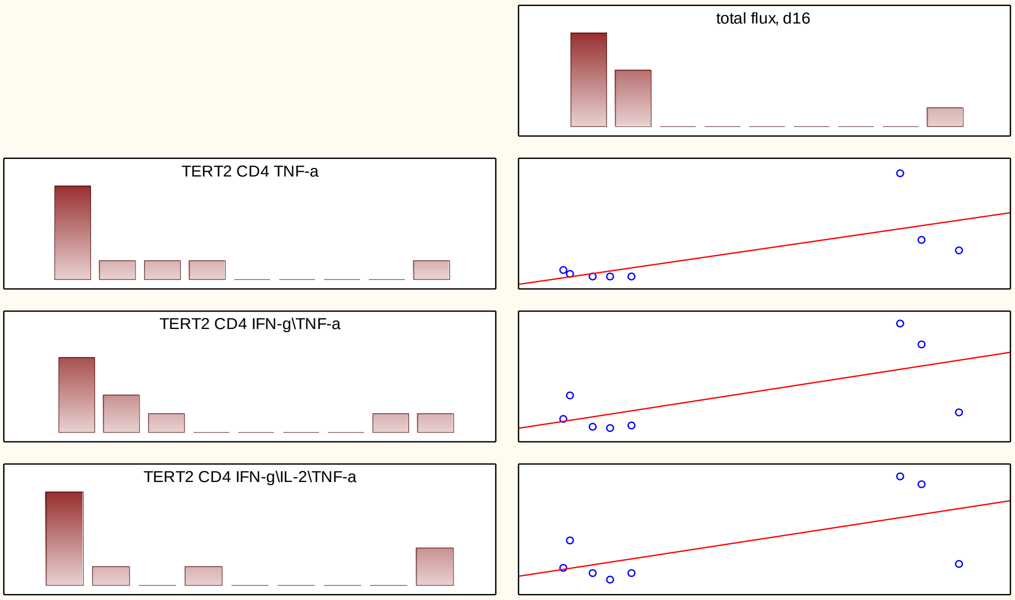

Supplementary Table S1. Primers used in PCR, RT-PCR and ddPCR assays.

Primer	Target Sequence Codes For	Direc-tion	Sequence 5′to 3′	Assay
TERT2	rt domain of TERT	FW	CTTTGGCGACATGGAGAACA	ddPCR
		RV	TTTGGCATGTGCAAGGTGAG	
b-act	Beta actin	FW	TTCACCTGCCCTGAGTGTTTC	ddPCR
		RV	TGAAGGTCTCAAACATGATCT GTAGA	
Mstn	Myostatine	FW	AAGACAACTTCTGCCAAGAG C	ddPCR
		RV	AATCTCCTTTCTCTGCTACTTA CAT	
rtTERT	rt domain of TERT	FW	AGAGCTTCAGGAGACAAGTG	PCR, RT-PCR
		RV	AGTCTCGTTCATGCTGATGG	
HPRT1	hypoxanthine phosphoribosyltransferase 1	FW	GGCCAGACTTTGTTGGATTT	PCR, RT-PCR
		RV	CAGATTCAACTTGCGCTCAT	

Supplementary Table S2. Thermal cycling protocol for ddPCR.

Temperature, °C	Time, Minutes	Ramp, °C/sec	Number of Cycles
95	5	2	1
95	0.5	2	35
59	1	2	
4	5	2	1
90	5	2	1
12	1	1	1

Supplementary Table S3. Correlations between % of CD4+ and CD8+ T cells of TERT/TERT_HA DNA-immunized mice capable to respond to in vitro stimulation with peptides derived from aa of TERT (TERT5 - TERT8; Table 1) by secretion of multiple cytokines, and capacity of respective mice to clear cells co-expressing TERT/TERT-HA and Luc assessed by *in vivo* imaging (BLI) in antigen challenge (see Materials and Methods for description). BLI data represent loss of the photon flux from the site of booster injection with TERT/TERT-HA and Luc DNA on days 7, 9 and 12 (d7, d9, d12) compared to day 1 (d1) after the boost, in %. Table shows Spearman correlation coefficients R, significant correlations ($p < 0.05$ and $p < 0.01$) are depicted in red.

Variable	Spearman Rank Order Correlations, p<0,05			Spearman Rank Order Correlations, p<0,01		
	BLI, % d7	BLI, % d9	BLI, % d12	BLI, % d7	BLI, % d9	BLI, % d12
CD4 IFN-g IL-2 TERT1	-0.351445	-0.371285	-0.382622	-0.351445	-0.371285	-0.382622
CD4 IFN-g TNF-a TERT1	-0.526725	-0.538301	-0.601971	-0.526725	-0.538301	-0.601971
CD4 IFN-g IL-2 TNF-a TERT1	-0.499570	-0.526493	-0.562390	-0.499570	-0.526493	-0.562390
CD8 IFN-g IL-2 TERT1	-0.362782	-0.393959	-0.427970	-0.362782	-0.393959	-0.427970
CD8 IFN-g TNF-a TERT1	-0.560312	-0.611250	-0.677169	-0.560312	-0.611250	-0.677169
CD8 IFN-g IL-2 TNF-a TERT1	-0.571365	-0.622219	-0.694014	-0.571365	-0.622219	-0.694014
CD4 IFN-g IL-2 TERT5	-0.517519	-0.562390	-0.634185	-0.517519	-0.562390	-0.634185
CD4 IFN-g TNF-a TERT5	-0.382831	-0.467556	-0.580522	-0.382831	-0.467556	-0.580522
CD4 IFN-g IL-2 TNF-a TERT5	-0.389107	-0.480108	-0.586798	-0.389107	-0.480108	-0.586798
CD8 IFN-g IL-2 TERT5	-0.571365	-0.622219	-0.694014	-0.571365	-0.622219	-0.694014
CD8 IFN-g TNF-a TERT5	-0.389107	-0.480108	-0.586798	-0.389107	-0.480108	-0.586798
CD8 IFN-g IL-2 TNF-a TERT5	-0.389107	-0.480108	-0.586798	-0.389107	-0.480108	-0.586798
CD4 IFN-g IL-2 TERT6	-0.491689	-0.581087	-0.561531	-0.491689	-0.581087	-0.561531
**CD4 IFN-g TNF-a TERT6**	-0.653723	-0.715184	-0.762677	-0.653723	-0.715184	-0.762677
**CD4 IFN-g IL-2 TNF-a TERT6**	-0.653723	-0.720772	-0.779439	-0.653723	-0.720772	-0.779439
CD8 IFN-g IL-2 TERT6	-0.491689	-0.581087	-0.561531	-0.491689	-0.581087	-0.561531
**CD8 IFN-g TNF-a TERT6**	-0.581087	-0.681660	-0.706803	-0.581087	-0.681660	-0.706803
**CD8 IFN-g IL-2 TNF-a TERT6**	-0.653723	-0.715184	-0.762677	-0.653723	-0.715184	-0.762677
CD4 IFN-g IL-2 TERT7	-0.459146	-0.555511	-0.510163	-0.459146	-0.555511	-0.510163
**CD4 IFN-g TNF-a TERT7**	-0.549842	-0.634869	-0.685885	-0.549842	-0.634869	-0.685885
**CD4 IFN-g IL-2 TNF-a TERT7**	-0.595190	-0.680217	-0.719896	-0.595190	-0.680217	-0.719896
CD8 IFN-g IL-2 TERT7	-0.481820	-0.578184	-0.544174	-0.481820	-0.578184	-0.544174
CD8 IFN-g TNF-a TERT7	-0.463267	-0.568681	-0.491008	-0.463267	-0.568681	-0.491008
CD8 IFN-g IL-2 TNF-a TERT7	-0.527168	-0.623532	-0.578184	-0.527168	-0.623532	-0.578184
CD4 IFN-g IL-2 TERT8	-0.519345	-0.613269	-0.599457	-0.519345	-0.613269	-0.599457
**CD4 IFN-g TNF-a TERT8**	-0.731327	-0.759029	-0.814433	-0.731327	-0.759029	-0.814433
**CD4 IFN-g IL-2 TNF-a TERT8**	-0.597849	-0.681660	-0.740327	-0.597849	-0.681660	-0.740327
CD8 IFN-g IL-2 TERT8	-0.488280	-0.593109	-0.609660	-0.488280	-0.593109	-0.609660
CD8 IFN-g TNF-a TERT8	-0.548970	-0.637247	-0.598626	-0.548970	-0.637247	-0.598626
**CD8 IFN-g IL-2 TNF-a TERT8**	-0.720006	-0.775179	-0.841387	-0.720006	-0.775179	-0.841387

Suppelmentary Table S4. Comparison of the profiles of in vitro cytokine production by CD4+ and CD8+ T cells of mice implanted with rtTERT expressing cell lines 4T1luc2_rtTERT_H9 (H9; n = 4) and 4T1luc2_rtTERT_C6 (C6; n = 4) and parental cell line (4T1luc2; n = 4) to stimulation with TERT2 presenting an autoepitope; TERT6, 8 and co-stimulation with TERT6 and TERT8. Cytokine production was assessed by multiparametric flow cytometry and presented as percent of mono-, di- and tri-cytokine-producing CD4+ and CD8+ T cells. Statistical analysis of integral profile of cytokine production was performed by Friedman ANOVA test and Kendall Coefficeint of concordance (Statistica AXA 10.0), $p < 0.01$ was considered as significant.

	Profile of cytokine production of CD4+ and CD8+ T cells to stimulation with TERT derived peptides					
	TERT2	TERT1	TERT6	TERT8	TERT6+8	PMA
CD4 T cells						
ANOVA Chi Sqr	**12,286**	**7,76**	**14**	**8**	**14**	**7,714**
Kendall Coefficient of Concordance	**0,877**	**0,554**	**1**	**0,571**	**1**	**0,551**
Average rank	**0,85**	**0,48**	**1**	**0,5**	**1**	**0,476**
p value	**0,002**	**0,02**	**0,0009**	**0,018**	**0,0009**	**0,02**
CD8 T cells						
ANOVA Chi Sqr	**12,286**	**10,571**	**10,64**	**10,571**	**11,629**	**8,857**
Kendall Coefficient of Concordance	**0,877**	**0,755**	**0,76**	**0,755**	**0,83**	**0,632**
Average rank	**0,857**	**0,714**	**0,72**	**0,714**	**0,802**	**0,571**
p value	**0,002**	**0,005**	**0,0049**	**0,005**	**0,003**	**0,012**

© 2020 by the authors. Submitted for possible open access publication under the terms and conditions of the Creative Commons Attribution (CC BY) license (http://creativecommons.org/licenses/by/4.0/).
